# Supplementary material for: Causal Relationship between Aging and Anorexia Nervosa: A White-Matter-Microstructure-Mediated Mendelian Randomization Analysis
Source: Biomedicines. 2024 Aug 16;12(8):1874. doi: 10.3390/biomedicines12081874 (PMC11351342; doi:10.3390/biomedicines12081874)
Supplement: Supplementary file 1 [file biomedicines-12-01874-s001.zip › Supplementary Figure/Figure S4/Singe SNP plot of MR analysis for TL to ubm-b-1461.pdf]

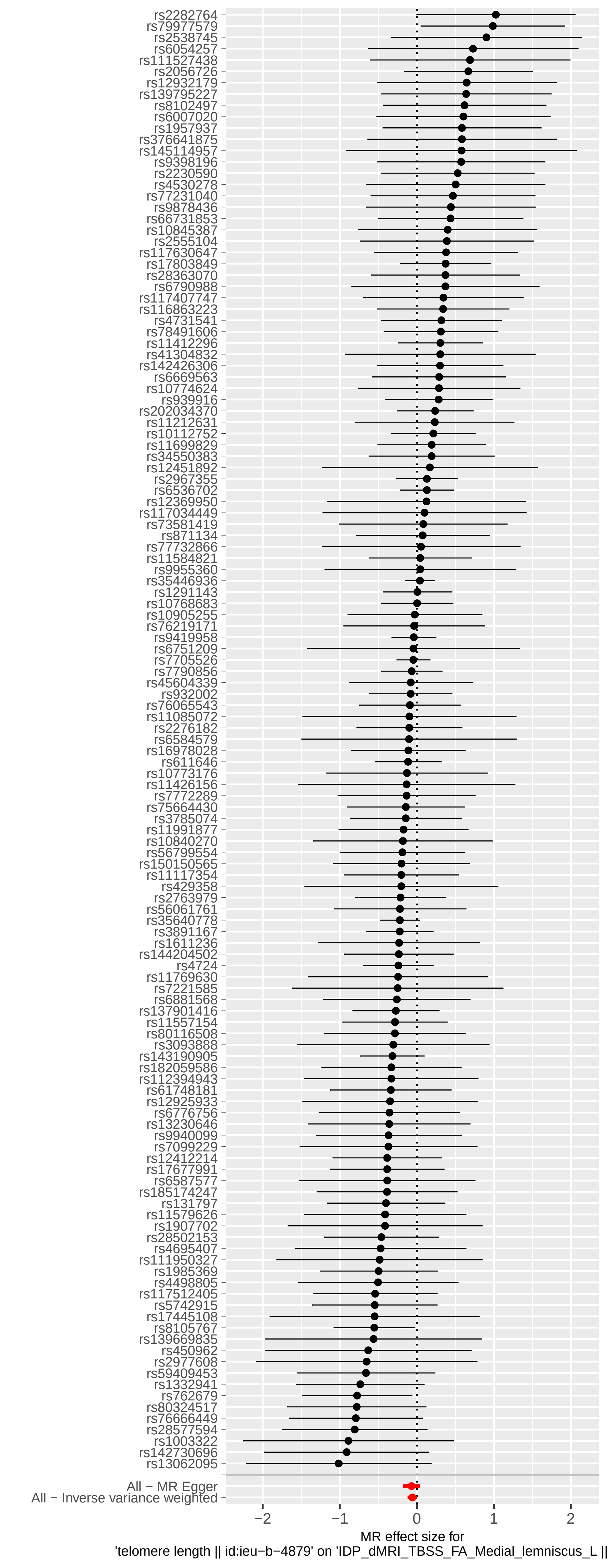
rs2282764
rs79977579
rs2538745
rs6054257
rs111527438
rs2056726
rs12932179
rs139795227
rs8102497
rs6007020
rs1957937
rs376641875
rs145114957
rs9398196
rs2230590
rs4530278
rs77231040
rs9878436
rs66731853
rs10845387
rs2555104
rs117630647
rs17803849
rs28363070
rs6790988
rs117407747
rs116863223
rs4731541
rs78491606
rs11412296
rs41304832
rs142426306
rs6669563
rs10774624
rs939916
rs202034370
rs11212631
rs10112752
rs11699829
rs34550383
rs12451892
rs2967355
rs6536702
rs12369950
rs117034449
rs73581419
rs871134
rs77732866
rs11584821
rs9955360
rs35446936
rs1291143
rs10768683
rs10905255
rs76219171
rs9419958
rs6751209
rs7705526
rs7790856
rs45604339
rs932002
rs76065543
rs11085072
rs2276182
rs6584579
rs16978028
rs611646
rs10773176
rs11426156
rs7772289
rs75664430
rs3785074
rs11991877
rs10840270
rs56799554
rs150150565
rs11117354
rs429358
rs2763979
rs56061761
rs35640778
rs3891167
rs1611236
rs144204502
rs4724
rs11769630
rs7221585
rs6881568
rs137901416
rs11557154
rs80116508
rs3093888
rs143190905
rs182059586
rs112394943
rs61748181
rs12925933
rs6776756
rs13230646
rs9940099
rs7099229
rs12412214
rs17677991
rs6587577
rs185174247
rs131797
rs11579626
rs1907702
rs28502153
rs4695407
rs111950327
rs1985369
rs4498805
rs117512405
rs5742915
rs17445108
rs8105767
rs139669835
rs450962
rs2977608
rs59409453
rs1332941
rs762679
rs80324517
rs76666449
rs28577594
rs1003322
rs142730696
rs13062095
All − MR Egger
All − Inverse variance weighted
−2
−1
0
1
2
MR effect size for
'telomere length || id:ieu−b−4879' on 'IDP_dMRI_TBSS_FA_Medial_lemniscus_L ||
